# The Role of the Systemic Immune-Inflammation Index in Predicting Postoperative Complications in Ovarian Cancer Patients: A Retrospective Cohort Study

**DOI:** 10.3390/cancers17071124

**Published:** 2025-03-27

**Authors:** Osman Köse, Elif Köse, Koray Gök, Mehmet Sühha Bostancı

**Affiliations:** 1 Department of Gynecologic Oncology, Sakarya University Faculty of Medicine, 54290 Adapazarı, Sakarya, Turkey; 2 Department of Public Health, Sakarya University Faculty of Medicine, 54290 Adapazarı, Sakarya, Turkey; elifkose@sakarya.edu.tr; 3 Department of Obstetrics and Gynecology, Cerrahpasa Faculty of Medicine, Istanbul University-Cerrahpasa, Kocamustafapasa, Fatih, 34098 Istanbul, Turkey; koray.gok@iuc.edu.tr; 4 Department of Obstetrics and Gynecology, Sakarya University Faculty of Medicine, 54290 Adapazarı, Sakarya, Turkey; msbostanci@sakarya.edu.tr

**Keywords:** ovarian cancer, complications, inflammation markers

## Abstract

Objective: Cytoreductive surgery (CRS) is the most important treatment method that increases survival in advanced-stage ovarian cancer (OC) patients. However, complications after CRS are seen as a significant cause of morbidity and mortality. Preoperative risk assessment of patients is of great importance. In recent years, inflammatory markers have been the subject of many studies evaluating malignancy and surgical outcomes. Ca125, Neutrophil–lymphocyte ratio (NLR), platelet–lymphocyte ratio (PLR), Monocyte–lymphocyte ratio (MLR), systemic inflammation index (SII), and systemic inflammatory response index (SIRI) stand out as prognostic and predictive tools in malignancies. This study aims to evaluate the preoperative inflammatory markers in patients who underwent CRS for advanced-stage epithelial ovarian cancer and to investigate the predictive power of postoperative complications. Materials and Methods: This retrospective study examines patients who underwent CRS due to advanced-stage epithelial OC at Sakarya University Training and Research Hospital between 2014 and 2023. Postoperative complications of the patients were graded according to the Clavien–Dindo classification (CDC); Ca125, NLR, PLR, SII, SIRI and MLR values were calculated using preoperative laboratory data, and the predictive values of inflammatory markers were analysed with ROC curves. Results: A significant relationship was found between complications with CDC ≥ 3 and NLR, PLR, MLR, SII, and SIRI. The AUC value of SII was calculated as 0.740 (*p* < 0.001), NLR as 0.719 (*p* = 0.001), PLR as 0.668 (*p* = 0.011), and SIRI as 0.651 (*p* = 0.022). SII stands out as the marker with the highest predictive power. SII is a strong marker in predicting postoperative complications, especially in advanced-stage OC patients. Conclusions: It was shown that preoperative inflammation markers may be an effective method for predicting postoperative complications in advanced-stage OC patients undergoing CRS. These findings may contribute to optimising surgical management and reducing complications. In future studies, these markers should be evaluated in groups with more patients, and their predictive power should be investigated.

## Simple Summary

Ovarian cancer (OC) is usually diagnosed at advanced stages, and cytoreductive surgery (CRS) is a crucial step in treatment to improve survival. However, this surgery can lead to serious complications that may negatively affect patients’ recovery and treatment outcomes. This study investigates whether systemic inflammation markers measured before surgery can predict complications following CRS. Data from 139 patients who underwent CRS at a single center between 2014 and 2023 were analysed, and postoperative complications were graded according to the Clavien–Dindo classification (CDC). A significant association was found between CDC grade ≥ 3 complications and markers such as NLR, PLR, SII, SIRI, and Ca125. Notably, SII emerged as a strong predictor of postoperative complications in advanced-stage OC patients. These findings could contribute to improving surgical management and reducing complications. Additionally, this approach may help identify patients preoperatively, potentially prioritising neoadjuvant chemotherapy to minimise complications.

## 1. Introduction

Ovarian cancer (OC) is the seventh most common type of cancer in women and is among the leading causes of death from reproductive cancer in women [1]. OC is usually diagnosed in advanced stages (FIGO stage III–IV) due to the lack of reliable screening methods to detect the early stages of the disease and the vagueness of clinical symptoms of the disease in the early stages. In addition to cytoreductive surgery (CRS), platinum-based chemotherapy [2] and maintenance treatments with drugs such as bevacizumab and poly (ADP-ribose) polymerase (PARP) inhibitors, which have become increasingly widespread in recent years, are used in patients diagnosed with advanced stages [3]. CRS, the most important factor that increases the patient’s survival time, involves the removal of all visible tumour tissues in gross terms and is the most important step of the treatment [4,5]. CRS is a comprehensive operation that includes extensive upper pelvic surgery such as diaphragm peeling and/or resection, partial liver resection, cholecystectomy, splenectomy, gastrectomy, distal pancreatectomy, as well as radical oophorectomy, hysterectomy, pelvic peritonectomy, bowel resections, and anastomosis during surgical procedures. A significant increase in life expectancy was observed in patients who achieved complete cytoreduction. A significant increase in life expectancy was observed in patients who achieved complete cytoreduction, while the rate of postoperative complications (33–64%) increased in parallel with the extent of the disease during surgery [6,7]. When complications that may occur after CRS are evaluated, we encounter infections, wound complications, venous thromboembolic events, organ failure, myocardial infarction, admission to the intensive care unit, and even surgery-related deaths. As a result of complications observed in the first 30 days after surgery, the initiation of chemotherapy may be delayed, or the dose of the chemotherapeutic agent to be administered may need to be reduced. Such negatively affecting conditions may directly negatively affect the survival rates of patients [8]. Complications after cytoreduction in advanced ovarian cancer are associated with factors such as age, general health status, low serum albumin levels (<3.5 g/dL), obesity, high peritoneal cancer index (≥20), cardiovascular comorbidities and surgical complexity. Careful assessment and management of these risk factors play an important role in reducing postoperative complications [9,10,11].

Recent studies have examined the possible relationship between cancer development and systemic inflammation and revealed that chronic systemic inflammation provides a favorable environment for tumour growth, angiogenesis and metastasis. Furthermore, inflammation-related markers such as the neutrophil-to-lymphocyte ratio (NLR), the platelet-to-lymphocyte ratio (PLR), the monocyte-to-lymphocyte ratio (MLR) and the systemic inflammation index (SII) are considered as potential biomarkers to predict poor prognosis, cancer progression and response to treatment in gynecologic solid tumours [12,13,14]. Similarly, studies in various malignancies such as breast cancer, gastric cancer, colon cancer and hepatocellular carcinoma show that these markers play an important role as independent predictive and prognostic factors [15,16,17,18]. In addition, these inflammation markers were also shown to predict early morbidity and mortality in cancers such as colorectal and gastric cancer [19,20].

In the literature, there is no study investigating the relationship between systemic inflammation markers and complications after ovarian cancer (OC) surgery. Based on this lack, this study aims to evaluate the role of easily measurable systemic inflammatory markers such as NLR, PLR, MLR, SII and SIRI in the preoperative period in the prediction of complications that may develop in patients undergoing advanced ovarian cancer surgery within the framework of the Clavien–Dindo Classification (CDC).

## 2. Material Method

This methodological study was conducted with the objective of investigating the relationship between preoperative inflammatory parameters and surgical complications of serous epithelial ovarian cancer. The study was performed by retrospectively reviewing the records of patients who underwent surgery for FIGO (International Federation of Gynecology and Obstetrics) Stage IIIc and IV ovarian, peritoneal and fallopian tube carcinoma at a tertiary care hospital between 1 January 2014 and 31 December 2023. A gynecologic oncologist evaluated all cases using a combination of diagnostic laparoscopy and open surgical techniques.

The inclusion criteria for the selection of patients were as follows: a diagnosis of FIGO Stage IIIc and IV epithelial ovarian, peritoneal and fallopian tube carcinoma, and surgical treatment. The exclusion criteria for the study were as follows: Patients who were referred for neoadjuvant chemotherapy, patients with Eastern Cooperative Oncology Group (ECOG) performance status > 3, patients who had received blood transfusions or bacterial/viral infections in the last two weeks, patients who were pregnant, patients with hematologic, thrombotic, rheumatologic or immunologic diseases, patients with severe liver or kidney disease, patients with a history of cancer, and patients receiving anticoagulant and/or antiplatelet therapy for venous or arterial thromboembolism were excluded from the study.

Accordingly, between 1 January 2014 and 31 December 2023, 228 patients were retrospectively reviewed through hospital records and a total of 89 patients were excluded and 139 patients were included in the study according to the exclusion criteria. The distribution of excluded patients is as follows:

- Patients with ECOG performance status > 3: 7 patients;
- Patients with known history of cancer: 12 patients (breast, colorectal, and thyroid cancers);
- Patients with a history of venous thromboembolism: 7 patients;
- Patients undergoing dialysis treatment: 2 patients;
- Patients referred for neoadjuvant chemotherapy: 61 patients.

In our routine clinical practice, patients referred for neoadjuvant chemotherapy have their peritoneal cancer index (PCI) evaluated primarily by computerised tomography (CT). Patients with a PCI ≥ 20 on CT are directly referred to neoadjuvant chemotherapy, while patients with a PCI < 20 undergo diagnostic laparoscopy to determine operative suitability. Patients in whom R0 resection is not possible after laparoscopy are also referred to neoadjuvant chemotherapy. All blood samples (hemogram and Ca125) are taken within two days before the operation. As part of our antibiotic protocol, intravenous (IV) antibiotics are started 30–60 min before surgery and cefazolin (first-generation cephalosporin) is generally preferred. If the patient is allergic, clindamycin is used as an alternative. If no complications (e.g., infection) develop during or after surgery, antibiotic treatment is not continued for more than 24 h.

Medical records, age, body mass index (BMI), American Society of Anesthesiologists (ASA) score, ECOG performance status, FIGO stage, preoperative albumin, serum cancer antigen (Ca125) levels, and complete blood count were obtained from the hospital data recording system. In addition, ascites, estimated blood loss, intraoperative blood transfusion, operative time, peritoneal cancer index (PCI), postoperative residual disease (RD) (RD 0: no residual disease, RD 1: 1–10 mm, RD 2: >10 mm), length of hospital stay, pathologic data, and finally complications within 30 days following surgery were retrospectively reviewed and recorded.

The primary objective of this study is to predict high-grade postoperative complications that develop within 30 days following surgery. These complications include the need for rehospitalisation, reoperation, cardiac arrest, myocardial infarction, stroke or cerebrovascular event, renal failure, venous thromboembolism, deep vein thrombosis, pulmonary embolism, sepsis, septic shock, pneumonia, and organ deep surgical site infection. The spectrum of complications is extensive, encompassing deep surgical site infection, ventilation for over 48 h, unplanned reintubation, bowel obstruction, anastomotic leak, ureteral obstruction, bladder fistula, ureteral fistula, or death. The Clavien–Dindo classification is a system employed globally to evaluate surgical complications in an objective and standardised manner. This classification system organises complications into five primary grades, ranging from mild (not requiring medical intervention) to life-threatening. Post-operative complications are categorised according to the Clavien–Dindo classification, with severe complications defined as ≥3a.As serious complications necessitate invasive treatment in patients, they are classified as follows: 3a: Intervention under local anaesthesia, 3b: Intervention under general anaesthesia, 4a: Single organ dysfunction, 4b: Multiple organ dysfunction, and 5: Death [21,22]. In this study, high-grade complications with grade ≥ 3 and above according to CDC were taken into consideration, as they play a critical role in determining treatment strategies after surgical intervention.

Various precautions were taken to minimise the risk of bias. During the data collection process, access to patient records was only performed by the study’s principal investigator and authorised personnel. The error rate was reduced by implementing a double-check mechanism during data collection. In addition, the inclusion and exclusion criteria used in patient selection were determined in advance and all patients were evaluated impartially according to these criteria. In order to minimise biases that may arise from the retrospective nature of the study, all data were directly obtained from the hospital information system and potential confounding factors were controlled with statistical methods during data analysis.

In this study, standard laboratory protocols were used for the measurement of preoperative inflammatory parameters. Hemogram measurements and neutrophil, lymphocyte, monocyte and platelet counts were recorded in thousand cells per microliter (K/µL) using automatic hematology analysers. Ca125 levels were measured by immunochemical methods and recorded in U/mL (units per milliliter). In addition, regular calibration of laboratory equipment was ensured. The following formulae are presented for the purpose of predicting severe complications. They are derived from inflammatory markers utilised in the study. When the abbreviations in the formulae are expressed as “N = Preoperative neutrophil value, M = Preoperative monocyte value, L = Preoperative lymphocyte value, P = Preoperative platelet value”, the following relationships can be established: NLR = N/L, MLR = M/L, PLR = P/L, SII = P × N/L, SIRI = M × N/L [23].

The study was conducted in accordance with the ethical standards set forth by Sakarya University Ethics Committee (no. 346 dated 13 November 2023) and with the informed consent of the patient.

### Statistical Analysis

In the study’s data evaluation, continuous parametric variables were presented as mean ± standard deviation (SD), and nonparametric variables were presented as median and interquartile range values. In contrast, categorical variables were shown as total numbers and percentages. Accordingly, the normality test of variables was evaluated using the Kolmogorov–Smirnov test and visual markers. After descriptive analysis of variables, the predictive power of Ca125, NLR, PLR, MLR, SII and SIRI values obtained in preoperative blood values for severe postoperative complications (CDC ≥ 3) was evaluated with ROC analysis. The predictive power of each marker for complications with CDC ≥ 3 was shown with the areas under the curve. The Youden index (J) was used to determine the optimal cut-off values of biomarkers. The Youden index is a widely preferred method to determine the most appropriate cut-off point that provides a balance between sensitivity and specificity. Its formula is as follows: J = Sensitivity + Specificity – 1.

The Youden index provides balance in the clinical decision-making process by considering both sensitivity and specificity. This index helps determine the point on the ROC curve that maximises the accuracy rate. The highest Youden index value represents the most clinically appropriate cut-off point of the biomarker [24]. The best cut-off values were calculated with the Youden Index, and sensitivity and specificity values were determined. Postoperative complications (CDC ≥ 3) aimed to be predicted by preoperatively obtained Ca125 value and NLR, PLR, MLR, SIRI, and SII ratios include grade 3—complications requiring surgical, endoscopic, or radiological intervention; grade 4—life-threatening complications (including central nervous system complications); and grade 5—mortality. The values of the two patient groups, patients at risk of mild complications and patients at risk of severe complications, were evaluated in terms of other preoperative clinical features with the obtained cut-off values. The Benjamini–Hochberg (BH) Correction is a statistical method used to control the false discovery rate (FDR) in biomarker studies. This method aims to reduce the number of false positive results in multiple hypothesis tests. By controlling the false discovery rate in multiple tests, the BH method reduces the number of false positive results, thus providing more reliable results. In addition, G-power analysis was used to evaluate the strength of the findings obtained in the retrospective study. While performing power analysis for each biomarker, AUC values higher than 0.5 and multiple testing corrections were taken into account (alpha values with Benjamini–Hochberg correction) [25]. The Chi-square or Mann–Whitney U tests were used during the evaluations according to the data characteristics. In addition, in order to predict severe postoperative complications, univariate and multivariate logistic regression analyses were used for the markers and clinical features obtained with the determined cut-off values. In multivariate logistic regression analysis, confounding variables of age and residual disease were controlled. Correlations of the continuous variable markers were examined with Spearmen correlation. ROC analysis was performed using the ‘pROC’ package in R. To ensure robustness, bootstrap resampling (n = 1000) was applied to estimate confidence intervals for the AUC and determine the optimal cutoff points. Data were analysed with IBM SPSS version 20.0. In all analyses, a *p*-value of less than 0.05 was considered statistically significant.

## 3. Results

**Figure 1:**
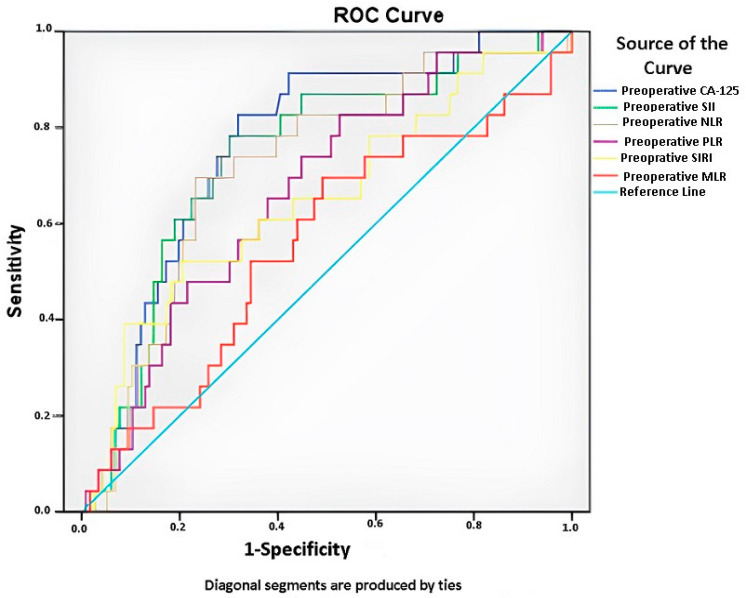
The predictive powers of different preoperative laboratory variables were compared with the AUC values according to the postoperative complications grade ≥ 3 CD classification. AUC: Area under the curve; CD: Clavien–Dindo; NLR: Neutrophil-to-lymphocyte ratio; PLR: Platelet-to-lymphocyte ratio; SII: Systemic immune inflammation index; SIRI: Systemic inflammatory response index; MLR: Monocyte–lymphocyte ratio.

The role and power of preoperatively examined blood parameter results and calculated ratios in predicting advanced complications postoperatively were evaluated and presented in Table 1 and Figure 1. Advanced complications were evaluated as a complication grade ≥ 3 according to the Clavian–Dindo classification. As seen in Table 1, AUC values for Preoperative Ca125 Value, Preoperative SII, Preoperative NLR, Preoperative PLR, Preoperative SIRI and Preoperative MLR were 0.766, 0.740, 0.719, 0.668, 0.651, and 0.559, respectively. According to the alpha value corrected by multiple test evaluation, preoperative Ca125, SII, NLR, PLR and SIRI values were found to be statistically significant predictors (*p* < 0.001, *p* < 0.001, *p* = 0.001, *p* = 0.011 and *p* = 0.022, respectively). According to the cut-off value of 838.5 U/mL, the sensitivity of preoperative Ca125 was 82.6% and the specificity was 68.1%; according to the cut-off value of 1151.2, the sensitivity of SII was 78.3% and the selectivity was 69.8%; according to the cut-off value of 4.1, the sensitivity of NLR was 69.6 and the selectivity was 76.7; according to the cut-off value of 167.4, the sensitivity of PLR was 82.6 and the selectivity was 47.4; and according to the cut-off value of 1.5, the sensitivity of SIRI was 60.9 and the selectivity was 63.8. Preoperative MLR was not found to be statistically significant (Table 1).

According to the results of the ROC analysis, in the group with a preoperative SII value below 1151.21, hemoglobin, hematocrit, and lymphocyte values were statistically higher. In contrast, leukocyte, platelet, and neutrophil values were statistically lower than in the group with 1151.21 and above. In the group with preoperative NLR values below 4.15, lymphocyte values were statistically higher than those with 4.15 and above; in contrast, leukocyte, platelet and neutrophil values were statistically lower, similar to SII. In the group with a preoperative PLR value below 167.38, hemoglobin, hematocrit, and lymphocyte values were statistically higher, similar to SII; in contrast, platelet and neutrophil values were statistically lower than those with 167.38 and above (Table 2).

According to the results of ROC analysis, there were statistically significant differences in the clinical variables of age, hypoalbuminemia, residual disease, ascites, PCI, bleeding amount, ASA, and operation time in the groups with preoperative Ca125 values below 838.5 and above 838 (*p* = 0.008, *p* = 0.002, *p* = 0.005, *p* < 0.001, *p* < 0.001, *p* = 0.008, *p* = 0.001, *p* < 0.001, respectively). Hypoalbuminemia significantly differed among the groups with NLR, PLR, SII, and SIRI cut-off values (*p* = 0.012, *p* < 0.001, *p* < 0.001, and *p* = 0.030, respectively). Similar to hypoalbuminemia, MLR levels differed among the groups according to the NLR, PLR, SII, and SIRI cut-off values. According to the cut-off values, MLR was also statistically significantly lower in groups with low NLR, PLR, SII and SIRI levels (*p* = <0.001, *p* < 0.001, *p* < 0.001, *p* < 0.001, respectively). Residual disease, PCI, and operation time variables differed in groups formed according to SII, Ca125, and NLR cut-off values. ASA variable differed in groups according to SII cut-off values and Ca125; ascites differed in groups according to NLR and Ca125 (Table 3).

The postoperative clinical evaluation and results according to the cut-off values of the markers are shown in Table 4. The duration of hospitalisation was statistically significantly longer in the groups with high Ca125, NLR, PLR, and SII values (*p* = 0.001, *p* = 0.002, *p* = 0.034, *p* = 0.003, respectively). The need for intensive care and the duration of subsequent intensive care unit stay were statistically significantly different in the groups determined according to Ca125 cut-off values. When all markers, including Ca125, NLR, PLR, SII, and SIRI, were compared according to the presence of complications, it was found that all of them provided statistical discrimination (*p* < 0.001, *p* < 0.001, *p* = 0.018, *p* < 0.001, *p* = 0.020, respectively). Statistically significant differences were found when the complication statuses were examined with CDC and mild and advanced (≥3a) complications in all markers (*p* < 0.001, *p* < 0.001, *p* = 0.008, *p* < 0.001, *p* = 0.034, respectively) (Table 4).

In the univariate analysis, age, hypoalbuminemia, residual disease, ascites, amount of bleeding, ASA score, operation time, hospital stay, intensive care unit stay, Ca125, NLR, PLR, SII, and SIRI categorical data were evaluated, and all were found to be statistically significantly associated with complications. Controlling for the confounders of age and residual disease, SII increased the risk by 8.49-fold (*p* = 0.016). Duration of operation and RD2 were also found to increase the risk of complications (*p* < 0.001, *p* = 0.045, respectively) (Table 5).

A weak positive correlation was found between Ca125 and Preoperative SII, Preoperative NLR, and Preoperative PLR. However, no statistically significant correlation was found between Preoperative SIRI and Preoperative MLR (Table 6).

## 4. Discussion

The present study investigates the role of preoperative inflammatory markers (NLR, PLR, SII and SIRI) in predicting postoperative complications in patients who underwent cytoreductive surgery for advanced epithelial ovarian cancer. The results demonstrate the efficacy of these inflammatory markers in predicting high-grade complications according to the CDC. Univariate analysis revealed that age, hypoalbuminemia, residual disease, amount of bleeding, duration of surgery, length of hospital stay, need for intensive care, Ca125 level and systemic inflammatory markers (NLR, PLR, SII, SIRI) were significantly associated with postoperative complications. Multivariate analysis identified the duration of surgery and SII as independent prognostic markers. These findings are critical for personalising treatment strategies and improving patient outcomes. The findings of this study may assist in the determination of optimal treatment approaches in the management of advanced ovarian cancer, offering the potential to increase survival rates and improve quality of life. Furthermore, they may serve as new prognostic markers to identify cases suitable for interval debulking surgery after neoadjuvant chemotherapy.

In a 2022 meta-analysis, it was reported that CRS increased the frequency of serious perioperative complications compared to standard surgery in patients with advanced-stage OC [6]. In studies conducted with standardised CDC, widely used to objectively and consistently evaluate postoperative complications, the prevalence of severe postoperative complications after CRS with CDC grade IIIa-V ranged from 4.9% to 29% [26,27,28]. In this study, the incidence of CDC grade ≥ IIIa was 16.5%, consistent with other studies. While mortality was found to be 0.7% in the current study, the average postoperative mortality after CRS for advanced-stage epithelial ovarian cancer was reported as 2.2–3.8% in a systematic review of studies conducted on community-based populations [27]. Achieving an optimal mass reduction rate with CRS in patients with advanced-stage OC significantly increases overall survival [22]. However, this situation increases the risk of both perioperative and postoperative complications, as surgical methods differ from each other. This shows that it is important to predict and effectively manage the complications that may arise after CRS.

In recent years, one of the most frequently studied prognostic factors in malignant diseases has been the systemic inflammatory status of patients. Tumour formation, progression and angiogenesis are closely related to the host immune system and homeostatic mechanisms [29,30].

Platelets promote the growth and invasion of tumour cells and the formation of metastatic foci through degranulation and release of proinflammatory cytokines (e.g., interleukin-6) and tumor growth factors (e.g., TGF-β) [31,32].

Similarly, high neutrophil counts are associated with increased tumour burden and cytokine release that facilitates cancer progression. In turn, lymphocytes have a critical role in inhibiting tumour spread and activating the immune response. Low lymphocyte levels in cancer patients have been associated with poor immune response and poor prognosis [33]. These findings underscore the pivotal function of inflammatory processes in cancer progression.

Numerous contemporary studies have further demonstrated the potential of inflammation markers to serve as prognostic indicators, capable of predicting the likelihood of postoperative complications [16,17,18]. Studies conducted with NLR, PLR, SII and SIRI have shown that they are associated with cancer progression, metastasis risk and poor prognosis. Studies conducted in the literature on postoperative complications and outcomes have recently begun to attract more attention. In a retrospective study by Chao-Yang Wang et al., 577 colon cancer patients were examined, and preoperative NLR value ≥ 2.66 was found to be associated with a higher incidence of postoperative symptomatic anastomotic leakage in elderly patients with intraoperative and postoperative blood transfusion within 2 days [17]. In a study of 662 colorectal cancer patients who underwent surgery between 2012 and 2014, it was reported that the combination of SII and tumour markers played a critical role in predicting postoperative complications and long-term outcomes [34]. Liung et al. associated SII with postoperative infectious complications and 5-year survival in a series of 440 colorectal patients [35]. Mungan et al. found that postoperative major complications (hospital and intensive care unit length of stay, anastomotic leakage, cardiorespiratory and renal problems) were significantly increased in 292 gastric cancer patients with preoperative PLR ≤ 0.55 and NLR > 3.92 [20]. In patients undergoing liver resection for HCC, it was found that those with high NLR values were an independent predictor of high-grade postoperative complications (peritonitis, intra-abdominal abscess, ileus, pneumonia, etc.) according to the CDC criteria [18]. In another study, it was suggested that preoperative SIRI, PLR, NLR and SII may better predict early postoperative recurrences in HCC patients [36]. However, further studies with larger data sets are required to better understand the changes of these biomarkers in the perioperative period and the threshold values that can be accepted as risk markers.

In this study, inflammation markers such as NLR ≥ 4.15, PLR ≥ 167.38, SII ≥ 1151.21 and SIRI ≥ 1.53 showed a significant association with complications ≥ 3 according to CDC criteria. The results of multivariate analysis, particularly the significance of SII, provide substantial evidence that preoperative inflammation markers have the potential to serve as a valuable tool in predicting serious postoperative complications.

Developing a model to predict postoperative complications and determine risk factors is very important. In a study conducted in Taiwan, a model was developed based on preoperative NLR values of 132 patients diagnosed with peritonitis carcinomatosis (60% OC). According to this model, it was found that high preoperative NLR values were associated with advanced age, increased PCI, incomplete cytoreduction, poor histological differentiation, significant complications and early mortality [37]. Similar findings were obtained in our study, and it was also shown that SII, SIRI and PLR may be important markers in predicting postoperative complications. In a study conducted on the postoperative course and usefulness of inflammatory markers after neoadjuvant chemotherapy, cytoreductive surgery and HIPEC in patients treated for ovarian peritoneal carcinomatosis, it was shown that NLR and CRP may play an important role in detecting infectious complications. However, the study was designed after surgery and performed on patients who underwent interval debulking surgery [38]. In contrast, the current study is based on preoperative prediction and offers a different approach. In a retrospective study evaluating the prognosis of 57 patients diagnosed with advanced ovarian cancer and undergoing cytoreductive surgery, it was shown that postoperative complications were correlated with inflammatory markers (MLR, PLR, NLR and SII) [12]. Similarly, while similar results were obtained with other inflammatory markers except MLR in this study, the size of the patient population and the evaluation of only the major complication group with CDC ≥ 3 may have been adequate in the lack of correlation with MLR.

Ca125 is a tumour marker often elevated in ovarian cancer. It was found to correlate with inflammatory markers like NLR and PLR, but the strength of these correlations can vary. For instance, Ca125 correlates positively with neutrophil count and NLR, suggesting a link between tumour activity and inflammation [39]. However, the correlation between Ca125 and these inflammatory markers is generally weak, indicating that they may reflect different aspects of disease pathology [12,40]. In this study, similar to the literature, correlations between Ca125 and other markers were found to be insignificant or weak.

Strengths of the study: Although many studies have emphasised the link between inflammation and malignancies, this is the first study to examine the association of these markers with complications after ovarian cancer surgery. In addition to demonstrating the importance of systemic inflammatory markers in predicting postoperative complications, it also contributes to the development of new approaches for risk management and treatment planning after surgery for advanced ovarian cancer.

The limitations of this study are as follows: Firstly, the generalisability of the findings is constrained due to the single-centre, retrospective study design and limited sample size. Secondly, the study focused on short-term outcomes and did not encompass information on long-term complications. Thirdly, only preoperative laboratory values were evaluated, and prognostic parameters such as CRP and albumin could not be analysed because CRP testing is not routine in patients undergoing CRS. Furthermore, low-grade complications were not included in the study, and biomarkers such as YKL-40, interleukin-10, interleukin-6 and tumour necrosis factor-α could not be evaluated due to cost and lack of widespread use. On the other hand, an important limitation of the study is the lack of external validation. Additional validation in different patient groups and multicenter studies is needed to increase the generalisability of our results.

## 5. Conclusions

According to the results of this study, preoperative inflammation markers in patients with ovarian cancer appear to be an effective way to predict postoperative complications. SII, NLR, PLR and SIRI were useful markers in determining the risk of complications. These findings suggest that high-risk patients can be identified preoperatively with these markers and patient management can be optimised. It is also possible that this approach may help select patients before surgery and prioritise neoadjuvant chemotherapy to reduce complications.

## Figures and Tables

**Table 1:** Comparison of the AUCs of preoperative laboratory values according to the postoperative complications grade ≥ 3 CD classification.

Items	Preoperative Ca125 Value	Preoperative SII	Preoperative NLR	Preoperative PLR	Preoperative SIRI	Preoperative MLR
AUC	0.766	0.739	0.718	0.652	0.652	0.560
SE	0.05	0.056	0.057	0.058	0.067	0.068
P	<0.001 *	<0.001 *	0.001 *	0.011 *	0.022 *	0.374
%95 CI	0.661–0.857	0.622–0.837	0.598–0.824	0.522–0.772	0.521–0.772	0.427–0.689
Associated criterion	838.5	1151.2	4.16	167.4	1.5	0.2
Sensitivity (%)	82.6	78.3	69.6	82.6	60.9	69.6
Specificity (%)	68.1	69.8	76.7	47.4	63.8	50.9
1 − β	0.98	0.96	0.94	0.76	0.70	0.04

* *p* < Benjamini–Hochberg Corrected alpha, AUC: Area under the curve; CD: Clavien–Dindo; NLR: Neutrophil-to-lymphocyte ratio; PLR: Platelet-to-lymphocyte ratio; SII: Systemic immune inflammation index, SIRI: Systemic inflammatory response index. MLR: Monocyte–lymphocyte ratio, CI: Confidence Interval.

**Table 2:** Distribution of some preoperative blood count values of patients according to Ca125, SII, NLR, PLR and SIRI cut-off values.

Variables	Total	Ca125		*p*	SII		*p*	NLR		*p*
		<838.50	≥838.50		<1151.21	≥1151.21		<4.15	≥4.15	
	Median [IQR]	Median [IQR]	Median [IQR]		Median [IQR]	Median [IQR]		Median [IQR]	Median [IQR]	
Hemoglobin (g/dL)	11.80 [2.1]	11.80 [2.00]	11.65 [2.35]	0.427	11.95 [1.92]	11.00 [2.51]	0.041	11.80 [1.80]	11.50 [3.18]	0.278
Hematocrit (%)	36.10 [6.00]	36.70 [5.70]	35.45 [6.27]	0.203	36.65 [4.45]	33.90 [7.50]	0.046	36.20 [5.30]	35.70 [8.20]	0.220
Monocyte (×10^3^/µL)	0.41 [0.24]	0.38 [0.25]	0.42 [0.25]	0.411	0.38 [0.24]	0.45 [0.24]	0.077	0.38 [0.24]	0.45 [0.24]	0.113
Leukocyte (×10^3^/µL)	7.11 [3.44]	6.82 [3.74]	7.37 [2.85]	0.125	6.33 [2.60]	8.41 [3.32]	<0.001	6.82 [2.90]	8.24 [4.92]	0.013
Platelet (×10^3^/µL)	284.0 [148.0]	266.0 [147.0]	328.5 [185.0]	<0.001	266.0 [114.2]	381.0 [199.0]	<0.001	281.0 [113.0]	351.0 [239.0]	0.005
Lymphocyte (×10^3^/µL)	1.59 [0.81]	1.64 [0.77]	1.50 [0.79]	0.230	1.75 [0.80]	1.34 [0.82]	<0.001	1.75 [0.76]	0.97 [0.78]	<0.001
Neutrophil (×10^3^/µL)	4.91 [2.99]	4.63 [3.16]	5.14 [2.99]	0.060	4.05 [1.99]	7.10 [3.07]	<0.001	4.31 [2.21]	7.74 [3.06]	<0.001
**Variables**	**Total**	**PLR**				***p***	**SIRI**			***p***
		**<167.38**		**≥167.38**			**<1.53**	**≥1.53**		
	**Median [IQR]**	**Median [IQR]**		**Median [IQR]**			**Median [IQR]**	**Median [IQR]**		
Hemoglobin (g/dL)	11.80 [2.10]	12.00 [2.00]		11.35 [2.20]		0.028	11.85 [1.82]	11.60 [2.76]		0.374
Hematocrit (%)	36.10 [6.00]	37.20 [4.50]		35.45 [6.05]		0.021	36.40 [5.30]	35.70 [7.80]		0.349
Monocyte (×10^3^/µL)	0.41 [0.24]	0.41 [0.25]		0.39 [0.25]		0.907	0.35 [0.17]	0.53 [0.26]		<0.001
Leukocyte (×10^3^/µL)	7.11 [3.44]	6.82 [3.16]		7.34 [3.52]		0.681	6.43 [2.71]	8.31 [3.45]		<0.001
Platelet (×10^3^/µL)	284.00 [148.00]	250.00 [94.00]		336.00 [188.75]		<0.001	274.50 [129.75]	332.00 [208.00]		0.001
Lymphocyte (×10^3^/µL)	1.59 [0.81]	1.99 [0.86]		1.35 [0.75]		<0.001	1.76 [0.78]	1.36 [0.81]		<0.001
Neutrophil (×10^3^/µL)	4.91 [2.99]	4.22 [2.86]		5.51 [3.13]		0.001	4.21 [2.24]	7.08 [3.74]		<0.001

IQR: Interquartile Range; NLR: Neutrophil-to-lymphocyte ratio; SII: Systemic immune inflammation index; SIRI: Systemic inflammatory response index; PLR: Platelet-to-lymphocyte ratio.

**Table 3:** Distribution of some preoperative clinical characteristics of patients according to Ca125, NLR, PLR, SII and SIRI cut-off values.

Variables	Total	Ca125		*p*	NLR		*p*	PLR		*p*
		<838.50	≥838.50		<4.15	≥4.15		<167.38	≥167.38	
	Median [IQR]	Median [IQR]	Median [IQR]		Median [IQR]	Median [IQR]		Median [IQR]	Median [IQR]	
Age	60.00 [16.00]	58.00 [16.00]	63.00 [15.00]	0.008	59.00 [16.00]	61.00 [12.00]	0.270	58.00 [16.00]	61.50 [15.75]	0.195
Hypoalbuminemia				0.002			0.012			<0.001
None	42 (30.2)	17 (20.5)	25 (44.6)		22 (23.2)	19 (44.2)		7 (11.9)	35 (43.8)	
Yes	97 (69.8)	66 (79.5)	31 (55.4)		73 (76.8)	24 (55.8)		52 (88.1)	45 (56.3)	
MLR	0.25 [0.20]	0.23 [0.22]	0.29 [0.17]	0.293	0.22 [0.12]	0.41 [0.18]	<0.001	0.20 [0.11]	0.34 [0.22]	<0.001
Residual disease				0.005			0.009			0.092
0	115(83.3)	75 (91.5)	40 (71.4)		84 (89.4)	30 (69.8)		53 (91.4)	62 (77.5)	
1	16 (11.6)	6 (7.3)	10 (17.9)		8 (8.5)	8 (18.6)		4 (6.9)	12 (15.0)	
2	7 (5.1)	1 (1.2)	6 (10.7)		2 (2.1)	5 (11.6)		1 (1.7)	6 (7.5)	
Acid (L)	1.25 [1.60]	0.95 [0.85]	2.15 [2.34]	<0.001	1.20 [1.10]	1.70 [4.00]	0.002	1.10 [10.50]	1.40 [2.51]	0.083
PCI				<0.001			0.005			0.116
<10	55 (39.6)	42 (50.6)	13 (23.2)		46 (48.4)	9 (20.9)		26 (44.1)	29 (36.3)	
10–15	48 (34.5)	30 (36.1)	18 (32.1)		30 (31.6)	17 (39.5)		23 (39.0)	25 (31.3)	
>15	36 (25.9)	11 (13.3)	25 (44.6)		19 (20.0)	17 (39.5)		10 (16.9)	26 (32.5)	
Amount of bleeding (L)	0.35 [0.25]	0.30 [0.20]	0.42 [0.30]	0.008	0.30 [0.17]	0.45 [0.35]	0.133	0.30 [0.20]	0.40 [0.35]	0.077
ASA				0.001			0.051			0.166
1	12 (8.6)	7(8.4)	5 (8.9)		11 (11.6)	1 (2.3)		6 (10.2)	6 (7.5)	
2	87 (62.6)	62 (74.7)	25 (44.6)		62 (65.3)	25 (58.1)		41 (69.5)	46 (57.5)	
3	40 (28.8)	14 (16.9)	26 (46.4)		22 (23.2)	17 (39.5)		12 (20.3)	28 (35.0)	
Operation time (min)	240.0 [80.0]	230.0 [50.0]	260.0 [80.0]	<0.001	230.0 [60.0]	260.0 [70.0]	0.008	230.0 [60.0]	240.0 [70.0]	0.140
**Variables**	**Total**		**SII**			***p***	**SIRI**			***p***
			**<1151.21**	**≥1151.21**			**<1.53**		**≥1.53**	
	**Median [IQR]**		**Median [IQR]**	**Median [IQR]**			**Median [IQR]**		**Median [IQR]**	
Age	60.00 [16.00]		59.50 [16.50]	61.00 [2.00]		0.374	59.00 [16.50]		61.00 [13.00]	0.447
Hypoalbuminemia						<0.001				0.030
None	42 (30.2)		16 (18.6)	26 (49.1)			19 (23.2)		23 (40.4)	
Yes	97 (69.8)		70 (81.4)	27 (50.9)			63 (76.8)		34 (59.6)	
MLR	0.25 [0.20]		0.22 [0.12]	0.37 [0.21]		<0.001	0.20 [0.08]		0.42 [0.16]	<0.001
Residual disease						0.041				0.248
0	115(83.3)		76 (89.4)	39 (73.6)			70 (85.4)		45 (80.4)	
1	16 (11.6)		7 (8.2)	9 (17.0)			10 (12.2)		6 (10.7)	
2	7 (5.1)		2 (2.4)	5 (9.4)			2 (2.4)		5 (8.9)	
Acid (L)	1.25 [1.60]		1.20 [1.10]	1.40 [3.00]		0.058	1.20 [1.25]		1.40 [2.60]	0.077
PCI						0.026				0.433
<10	55 (39.6)		41 (47.7)	14 (26.4)			36 (43.9)		19 (33.3)	
10–15	48 (34.5)		28 (32.6)	20 (37.7)			27 (32.9)		21 (36.9)	
>15	36 (25.9)		17 (19.8)	19 (35.8)			19 (23.2)		17 (29.8)	
Amount of bleeding (L)	0.35 [0.25]		0.30 [0.15]	0.40 [0.35]		0.135	0.30 [0.23]		0.35 [0.32]	0.704
ASA						0.018				0.092
1	12 (8.6)		10 (11.6)	2 (3.8)			7 (8.5)		5 (8.8)	
2	87 (62.6)		58 (67.4)	29 (54.7)			57 (69.5)		30 (52.6)	
3	40 (28.8)		18 (20.9)	22 (41.5)			18 (22.0)		22 (38.6)	
Operation time	240.0 [80.0]		230.0 [60.0]	250.0 [75.0]		0.019	235.0 [70.0]		250.0 [75.0]	0.278

IQR: Interquartile Range; NLR: Neutrophil-to-lymphocyte ratio; PLR: Platelet-to-lymphocyte ratio; SII: Systemic immune inflammation index; SIRI: Systemic inflammatory response index.

**Table 4:** Distribution of some postoperative clinical features and complications of patients according to Ca125, NLR, PLR, SII and SIRI cut-off values.

Variables	Total	Ca125		*p*	NLR		*p*	PLR		*p*
		<838.50	≥838.50		<4.15	≥4.15		<167.38	≥167.38	
	Median [IQR]	Median [IQR]	Median [IQR]		Median [IQR]	Median [IQR]		Median [IQR]	Median [IQR]	
Duration of hospital stay	60.0 [3.0]	6.0 [3.0]	8.0 [4.0]	0.001	6.0 [3.0]	8.0 [4.0]	0.002	6.0 [3.0]	7.0 [4.0]	0.034
ICU				0.028			0.815			0.662
Yes	21 (15.1)	8 (9.6)	13 (23.2)		14 (14.7)	7 (16.3)		8 (13.6)	13 (16.3)	
No	118 (84.9)	75 (90.4)	43 (76.8)		81 (85.3)	36 (83.7)		51 (86.4)	67 (83.3)	
Intensive care unit length of stay	0 [5.0]	0 [5.0]	0 [5.0]	0.026	0 [4.0]	0 [5.0]	0.713	0 [4.0]	0 [5.0]	0.622
Complications				<0.001 *			<0.001 *			0.018*
None	117 (84.2)	79 (95.2)	38 (67.9)		88 (92.6)	28 (65.1)		55(93.2)	62 (77.5)	
Cardiovascular	4 (2.9)	-	4 (7.19		-	4 (9.3)		-	4 (5.0)	
Surgery site	4 (2.9)	1 (1.2)	3 (5.4)		-	4 (9.3)		-	4 (5.0)	
Respiratory	4 (2.9)	1(1.2)	3 (5.4)		2 (2.1)	2 (4.7)		1 (1.7)	3 (3.8)	
Infections	4 (2.9)	-	4 (7.1)		3 (3.2)	1 (2.3)		3 (5.1)	1 (1.3)	
Gastrointestinal	3 (2.2)	1 (1.2)	2 (3.6)		2 (2.1)	1 (2.3)		-	3 (3.8)	
Anastomotic Leaks	3 (2.2)	1 (1.2)	2 (3.6)		-	3 (7.0)		-	3 (3.8)	
Complication				<0.001			<0.001			0.008
0–2	116 (83.5)	79 (95.2)	37 (66.1)		88 (92.6)	27 (62.8)		55 (93.2)	61 (76.3)	
≥3a	23 (16.50)	4 (4.8)	19 (33.9)		7 (7.4)	16 (37.2)		4 (6.8)	19 (23.8)	
Operation-related death				0.222						0.389
Yes	1(0.7)	-	1 (1.8)		-	1 (2.3)	0.136	-	1 (1.3)	
No	138 (99.3)	83 (100.0)	55 (98.2)		95 (100.0)	42 (97.7)		59 (100.0)	79 (98.8)	
**Variables**	**Total**	**SII**				***p***	**SIRI**			***p***
		**<1151.21**		**≥1151.21**			**<1.53**		**≥1.53**	
	**Median [IQR]**	**Median [IQR]**		**Median [IQR]**			**Median [IQR]**		**Median [IQR]**	
Duration of hospital stay	60.0 [3.0]	6.0 [3.0]		8.0 [4.50]		0.003	6.0 [3.0]		7.0 [4.0]	0.396
ICU						0.331				0.082
Yes	21 (15.1)	11 (12.8)		10 (18.9)			16 (19.5)		5 (8.8)	
No	118 (84.9)	75 (87.2)		43 (81.1)			66 (80.5)		52 (91.2)	
Intensive care unit length of stay	0 [5.0]	0 [4.0]		0 [5.0]		0.319	0 [4.0]		0 [5.0]	1.000
Complications						<0.001 *				0.020 *
None	117(84.2)	81 (94.2)		36 (67.9)			73 (89.0)		44 (77.2)	
Cardiovascular	4 (2.9)	-		4 (7.5)			1 (1.2)		3 (5.3)	
Surgery site	4 (2.9)	-		4 (7.5)			-		4 (7.0)	
Respiratory	4 (2.9)	2 (2.3)		2 (3.8)			3 (3.7)		1 (1.8)	
Infections	4 (2.9)	2 (2.3)		2 (3.8)			3 (3.7)		1(1.8)	
Gastrointestinal	3 (2.2)	1 (81.2)		2 (3.8)			2 (2.4)		1 (1.89)	
Anastomotic Leaks	3 (2.2)	-		3 (5.7)			-		3 (5.3)	
Complication						<0.001				0.034
0–2	116 (83.5)	81 (94.2)		35 (66.0)			73 (89.0)		43 (75.4)	
≥3a	23 (16.50)	5 (5.8)		18 (34.0)			9 (11.0)		14 (24.6)	
Operation-related death										
Yes	1 (0.7)	-		1 (1.8)		0.201	-		56 (98.2)	0.229
No	138 (99.3)	86 (100.0)		52 (98.1)			82 (100.0)		1 (1.8)	

* Chi-square Exact test T; NLR: Neutrophil-to-lymphocyte ratio; PLR: Platelet-to-lymphocyte ratio; SII: Systemic immune inflammation index; SIRI: Systemic inflammatory response index.

**Table 5:** Univariate and multivariate logistic regression analyses of postoperative clinical features and complications in ovarian cancer patients.

Variables	Univariate Analysis		Multivariate Analysis	
	RR (%95 CI)	*p*	RR (%95 GA)	*p*
Age	1.045 (1.000–1.091)	0.048	0.993 (0.911–1.082)	0.870
Hypoalbuminemia				
(Yes)	6.181 (2.366–16.143)	<0.001	-	-
Residual disease (RD)				
0	1	<0.001	1	0.134
1	5.673 (1.730–18.603)	0.004	1.428 (0.225–9.078)	0.706
2	56.727 (6.246–515.212)	<0.001	16.430 (1.065–253.369)	0.045
Acid (Yes)	1.001 (1.001–1.001)	<0.001	-	-
PCI		<0.001	-	-
≤15	1			
>15	19.600 (6.453–59.533)			
Amount of bleeding	1.007 (1.005–1.010)	<0.001	-	-
ASA		<0.001	-	-
1–2	1			
3	21.488 (6.621–69.738)			
Operation time (dk)	1.056 (1.033–1.080)	<0.001	1.052 (1.024–1.080)	<0.001
Duration of hospital stay (day)	5.865 (2.493–13.797)	<0.001	-	-
ICU (Yes)	17.550 (5.881–52.373)	<0.001	-	-
ICU length of stay (day)	3.717 (2.192–6.303)	<0.001	-	-
Ca125				
≥838.50	10.142 (3.222–31.926)	<0.001	1.901 (0.386–9.361)	0.429
NLR				
≥4.15	7.450 (2.776–19.995)	<0.001	-	-
PLR				
≥167.38	4.283 (1.372–13.366)	0.012	-	-
SII				
≥1151.21	8.331 (2.866–24.221)	<0.001	8.498 (1.498–48.200)	0.016
SIRI				
≥1.53	2.641 (1.054–6.615)	0.038	-	-

CI: Confidence Interval; ICU: Intensive care unit; NLR: Neutrophil-to-lymphocyte ratio; PLR: Platelet-to-lymphocyte ratio; SII: Systemic immune inflammation index; SIRI: Systemic inflammatory response index.

**Table 6:** Correlations of Ca125 with other systemic inflammatory indices.

Variables	Preoperative Ca125	
	r	*p*
Preoperative SII	0.263	0.002
Preoperative NLR	0.217	0.010
Preoperative PLR	0.282	0.001
Preoperative SIRI	0.119	0.164
Preoperative MLR	0.086	0.313

NLR: Neutrophil-to-lymphocyte ratio; PLR: Platelet-to-lymphocyte ratio; SII: Systemic immune inflammation index; SIRI: Systemic inflammatory response index; MLR: Monocyte–lymphocyte ratio.

## Data Availability

Data presented in this study are available from the corresponding author upon reasonable request.

## References

[1] Bryant A., Hiu S., Kunonga P.T., Gajjar K., Craig D., Vale L., Winter-Roach B.A., Elattar A., Naik R. (2022). Impact of residual disease as a prognostic factor for survival in women with advanced epithelial ovarian cancer after primary surgery. Cochrane Database Syst. Rev..

[2] Lee Y.J., Lee J.Y., Nam E.J., Kim S.W., Kim S., Kim Y.T. (2020). Rethinking radical surgery in interval debulking surgery for advanced-stage ovarian cancer patients undergoing neoadjuvant chemotherapy. J. Clin. Med..

[3] Cortez A.J., Tudrej P., Kujawa K.A., Lisowska K.M. (2018). Advances in ovarian cancer therapy. Cancer Chemother. Pharmacol..

[4] Aletti G.D., Dowdy S.C., Gostout B.S., Jones M.B., Stanhope C.R., Wilson T.O., Podratz K.C., Cliby W.A. (2006). Aggressive surgical effort and improved survival in advanced-stage ovarian cancer. Obstet. Gynecol..

[5] Chi D.S., Eisenhauer E.L., Zivanovic O., Sonoda Y., Abu-Rustum N.R., Levine D.A., Guile M.W., Bristow R.E., Aghajanian C., Barakat R.R. (2009). Improved progression-free and overall survival in advanced ovarian cancer as a result of a change in surgical paradigm. Gynecol. Oncol..

[6] Kengsakul M., Nieuwenhuyzen-de Boer G.M., Udomkarnjananun S., Kerr S.J., Niehot C.D., van Beekhuizen H.J. (2022). Factors predicting postoperative morbidity after cytoreductive surgery for ovarian cancer: A systematic review and meta-analysis. J. Gynecol. Oncol..

[7] Aletti G.D., Eisenhauer E.L., Santillan A., Axtell A., Aletti G., Holschneider C., Chi D.S., Bristow R.E., Cliby W.A. (2011). Identification of patient groups at highest risk from traditional approach to ovarian cancer treatment. Gynecol. Oncol..

[8] Kumar A., Janco J.M., Mariani A., Bakkum-Gamez J.N., Langstraat C.L., Weaver A.L., McGree M.E., Cliby W.A. (2016). Risk-prediction model of severe postoperative complications after primary debulking surgery for advanced ovarian cancer. Gynecol. Oncol..

[9] Günakan E., Tohma Y.A., Tunç M., Akıllı H., Şahin H., Ayhan A. (2020). Factors associated with surgical morbidity of primary debulking in epithelial ovarian cancer. Obstet. Gynecol. Sci..

[10] Zighelboim I., Kizer N., Taylor N.P., Case A.S., Gao F., Thaker P.H., Rader J.S., Massad L.S., Mutch D.G., Powell M.A. (2010). “Surgical Apgar Score” predicts postoperative complications after cytoreduction for advanced ovarian cancer. Gynecol. Oncol..

[11] Bacalbasa N., Balescu I., Dimitriu M., Iliescu L., Diaconu C., Dima S., Vilcu M., Brezean I. (2020). The Influence of the Preoperative Status on the Risk of Postoperative Complications After Cytoreductive Surgery for Advanced-stage Ovarian Cancer. In Vivo.

[12] Balescu I., Eftimie M., Petrea S., Diaconu C., Gaspar B., Pop L., Varlas V., Hasegan A., Martac C., Bolca C. (2024). Prognostic significance of preoperative inflammation markers on the long-term outcomes in peritoneal carcinomatosis from ovarian cancer. Cancers.

[13] Leng J., Wu F., Zhang L. (2022). Prognostic significance of pretreatment neutrophil-to-lymphocyte ratio, platelet-to-lymphocyte ratio, or monocyte-to-lymphocyte ratio in endometrial neoplasms: A systematic review and meta-analysis. Front. Oncol..

[14] Guo J., Lv W., Wang Z., Shang Y., Yang F., Zhang X., Xiao K., Zhang S., Pan X., Han Y. (2023). Prognostic value of inflammatory and nutritional markers for patients with early-stage poorly-to moderately-differentiated cervical squamous cell carcinoma. Cancer Control..

[15] Yuce E., Karakullukcu S., Bulbul H., Alandag C., Saygin I., Kavgaci H. (2023). The effect of the change in hemoglobin-albumin-lymphocyte-platelet scores occurring with neoadjuvant chemotherapy on clinical and pathological responses in breast cancer. Bratisl. Lek. Listy..

[16] Chen X., Zhang Y., Liu Z., Song J., Li J. (2024). The inflammation score predicts the prognosis of gastric cancer patients undergoing Da Vinci robot surgery. J. Robot. Surg..

[17] Wang C.Y., Li X.L., Ma X.L., Yang X.F., Liu Y.Y., Yu Y.J. (2024). Preoperative neutrophil-to-lymphocyte ratio predicts symptomatic anastomotic leakage in elderly colon cancer patients: Multicenter propensity score-matched analysis. World J. Gastrointest. Surg..

[18] Wu H.L., Liu H.Y., Liu W.C., Hou M.C., Tai Y.H. (2022). A predictive model incorporating inflammation markers for high-grade surgical complications following liver resection for hepatocellular carcinoma. J. Chin. Med. Assoc..

[19] Shevchenko I., Grigorescu C.C., Serban D., Cristea B.M., Simion L., Gherghiceanu F., Costea A.C., Dumitrescu D., Alius C., Tudor C. (2024). The value of systemic inflammatory indices for predicting early postoperative complications in colorectal cancer. Medicina.

[20] Mungan İ., Dicle Ç.B., Bektaş Ş., Sarı S., Yamanyar S., Çavuş M., Turan S., Bostancı E.B. (2020). Does the preoperative platelet-to-lymphocyte ratio and neutrophil-to-lymphocyte ratio predict morbidity after gastrectomy for gastric cancer?. Mil. Med. Res..

[21] Dindo D., Demartines N., Clavien P.A. (2004). Classification of surgical complications: A new proposal with evaluation in a cohort of 6336 patients and results of a survey. Ann. Surg..

[22] Elyashiv O., Graham R., Counsell N., Jayanth N., Berg L., Howard K., Gleeson J.T., Doufekas K., Macdonald N.D., Ledermann J.A. (2024). Survival outcomes following cytoreductive surgery in advanced ovarian cancer patients: Prognosis is better predicted by the completeness of resection than by disease stage. Int. J. Gynecol. Cancer..

[23] Wang R.H., Wen W.X., Jiang Z.P., Du Z.P., Ma Z.H., Lu A.L., Li H.P., Yuan F., Wu S.B., Guo J.W. (2023). The clinical value of neutrophil-to-lymphocyte ratio (NLR), systemic immune-inflammation index (SII), platelet-to-lymphocyte ratio (PLR) and systemic inflammation response index (SIRI) for predicting the occurrence and severity of pneumonia in patients with intracerebral hemorrhage. Front Immunol..

[24] Fluss R., Faraggi D., Reiser B. (2005). Estimation of the Youden Index and its associated cutoff point. Biom. J..

[25] Ferreira J. (2007). The Benjamini-Hochberg Method in the Case of Discrete Test Statistics. Int. J. Biostat..

[26] Barakat P., Nieroda C., Diaz-Montes T., Gushchin V. (2023). Peritoneal metastases from rare ovarian cancer treated with cytoreductive surgery and hyperthermic intraperitoneal chemotherapy (CRS/HIPEC). Pleura Peritoneum..

[27] Narasimhulu D.M., Thannickal A., Kumar A., Weaver A.L., McGree M.E., Langstraat C.L., Cliby W.A. (2021). Appropriate triage allows aggressive primary debulking surgery with rates of morbidity and mortality comparable to interval surgery after chemotherapy. Gynecol. Oncol..

[28] Inci M.G., Rasch J., Woopen H., Mueller K., Richter R., Sehouli J. (2021). ECOG and BMI as preoperative risk factors for severe postoperative complications in ovarian cancer patients: Results of a prospective study (RISC-GYN-trial). Arch. Gynecol. Obstet..

[29] Savant S.S., Sriramkumar S., O’Hagan H.M. (2018). The Role of Inflammation and Inflammatory Mediators in the Development, Progression, Metastasis, and Chemoresistance of Epithelial Ovarian Cancer. Cancers.

[30] Stoiber D., Assinger A. (2020). Platelet-Leukocyte Interplay in Cancer Development and Progression. Cells.

[31] Browning L., Patel M.R., Horvath E.B., Tawara K., Jorcyk C.L. (2018). IL-6 and ovarian cancer: Inflammatory cytokines in promotion of metastasis. Cancer Manag. Res..

[32] Hao Y., Baker D., Ten D.P. (2019). TGF-β-Mediated Epithelial-Mesenchymal Transition and Cancer Metastasis. Int. J. Mol. Sci..

[33] Ménétrier-Caux C., Ray-Coquard I., Blay J.Y., Caux C. (2019). Lymphopenia in Cancer Patients and its Effects on Response to Immunotherapy: An opportunity for combination with Cytokines?. J. Immunother. Cancer.

[34] Xie H., Yuan G., Huang S., Kuang J., Yan L., Ruan G., Tang S., Gan J. (2020). The prognostic value of combined tumor markers and systemic immune-inflammation index in colorectal cancer patients. Langenbecks Arch. Surg..

[35] Feng L., Xu R., Lin L., Liao X. (2022). Effect of the systemic immune-inflammation index on postoperative complications and the long-term prognosis of patients with colorectal cancer: A retrospective cohort study. J. Gastrointest. Oncol..

[36] Wenpei G., Yuan L., Liangbo L., Jingjun M., Bo W., Zhiqiang N., Yijie N., Lixin L. (2023). Predictive value of preoperative inflammatory indexes for postoperative early recurrence of hepatitis B-related hepatocellular carcinoma. Front. Oncol..

[37] Hung H.C., Hsu P.J., Chang T.C., Chou H.H., Huang K.G., Lai C.H., Lee C.W., Yu M.C., You J.F., Hsu J.T. (2022). Neutrophil-to-lymphocyte-ratio-based perioperative prognosis prediction model on early mortality after cytoreductive surgery with hyperthermic intraperitoneal chemotherapy. Asian J. Surg..

[38] Medina Fernández F.J., Muñoz-Casares F.C., Arjona-Sánchez A., Casado-Adam A., Gómez-Luque I., Garcilazo Arismendi D.J., Thoelecke H., Rufián P.S., Briceño D.J. (2015). Postoperative time course and utility of inflammatory markers in patients with ovarian peritoneal carcinomatosis treated with neoadjuvant chemotherapy, cytoreductive surgery, and HIPEC. Ann. Surg. Oncol..

[39] Williams K.A., Labidi-Galy S.I., Terry K.L., Vitonis A.F., Welch W.R., Goodman A., Cramer D.W. (2014). Prognostic significance and predictors of the neutrophil-to-lymphocyte ratio in ovarian cancer. Gynecol Oncol..

[40] Baert T., Van Camp J., Vanbrabant L., Busschaert P., Laenen A., Han S., Van Nieuwenhuysen E., Vergote I., Coosemans A. (2018). Influence of CA125, platelet count and neutrophil to lymphocyte ratio on the immune system of ovarian cancer patients. Gynecol. Oncol..

